# Supplementary material for: Rapid Identification by Resequencing-Based QTL Mapping of a Novel Allele RGA1-FH Decreasing Grain Length in a Rice Restorer Line ‘Fuhui212’
Source: Int J Mol Sci. 2023 Jun 28;24(13):10746. doi: 10.3390/ijms241310746 (PMC10341677; doi:10.3390/ijms241310746)
Supplement: Supplementary file 1 [file ijms-24-10746-s001.zip › ijms-2451841-supplementary.pdf]

Supplementary Files

Figure S1. Distribution of variant markers used for QTL mapping. Grey bars represent putative centromeres.

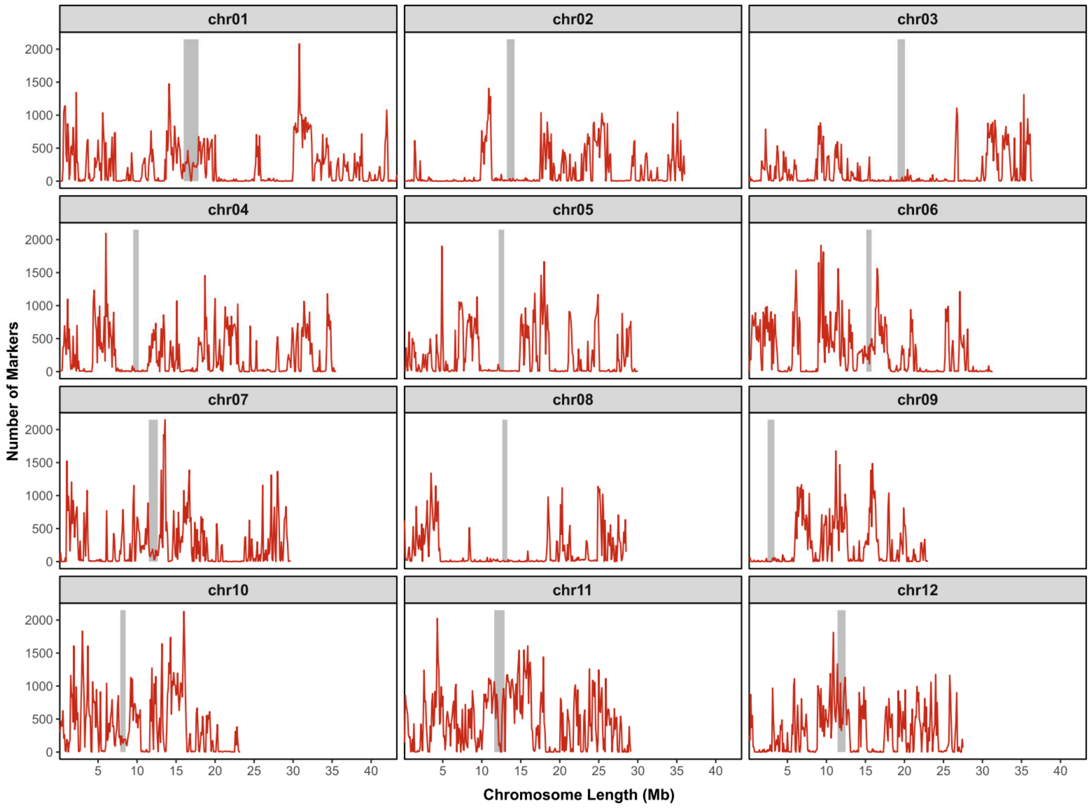

Figure S2. Distribution of LOD scores from QTL mapping. Red dashed line: $p < 0.05$; blue dashed line: $p < 0.01$.

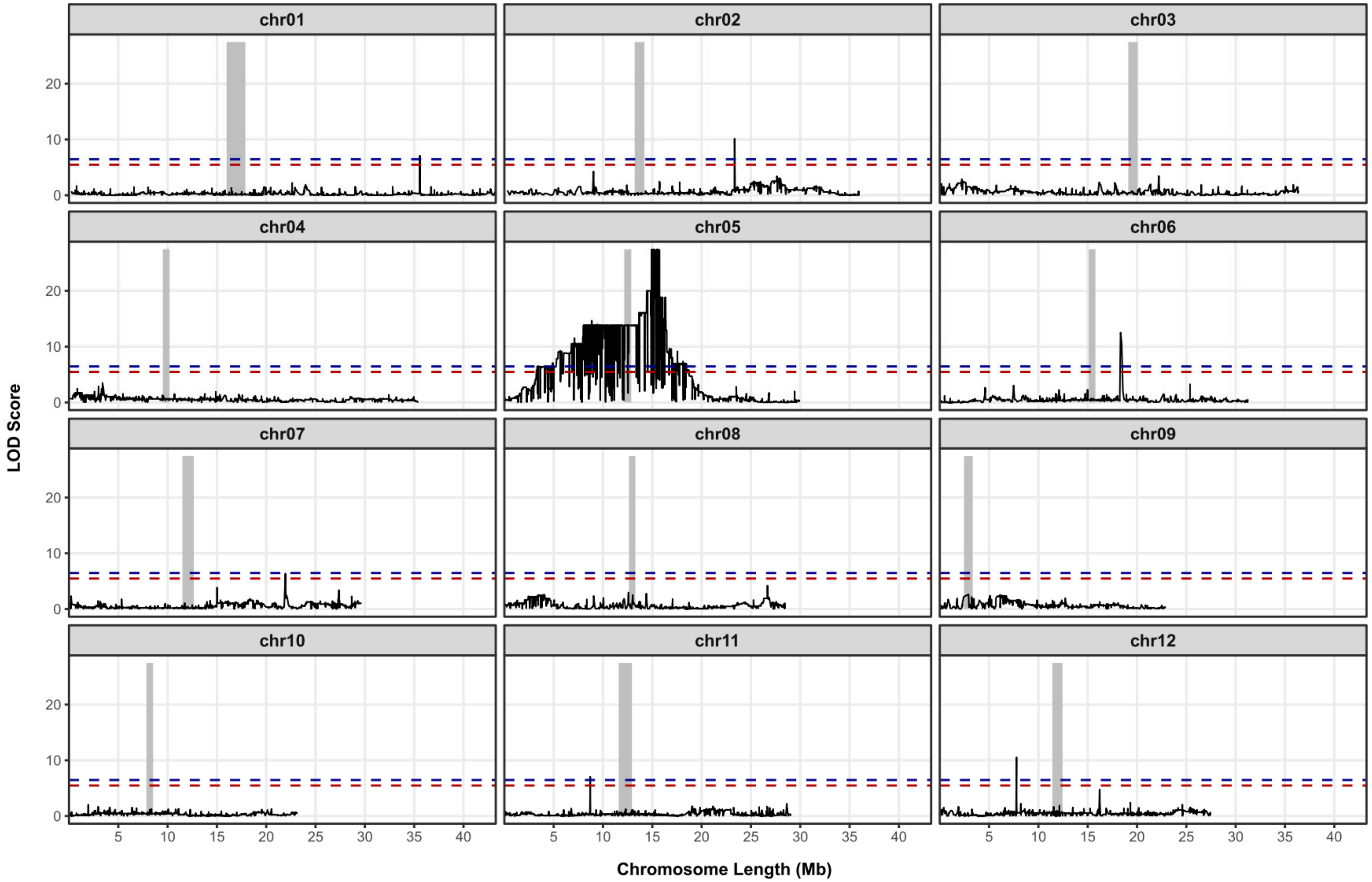

Figure S3. Predicted effects of 4,851 co-segregated variants within chromosome 5 region. Variant numbers for each category are given in parentheses.

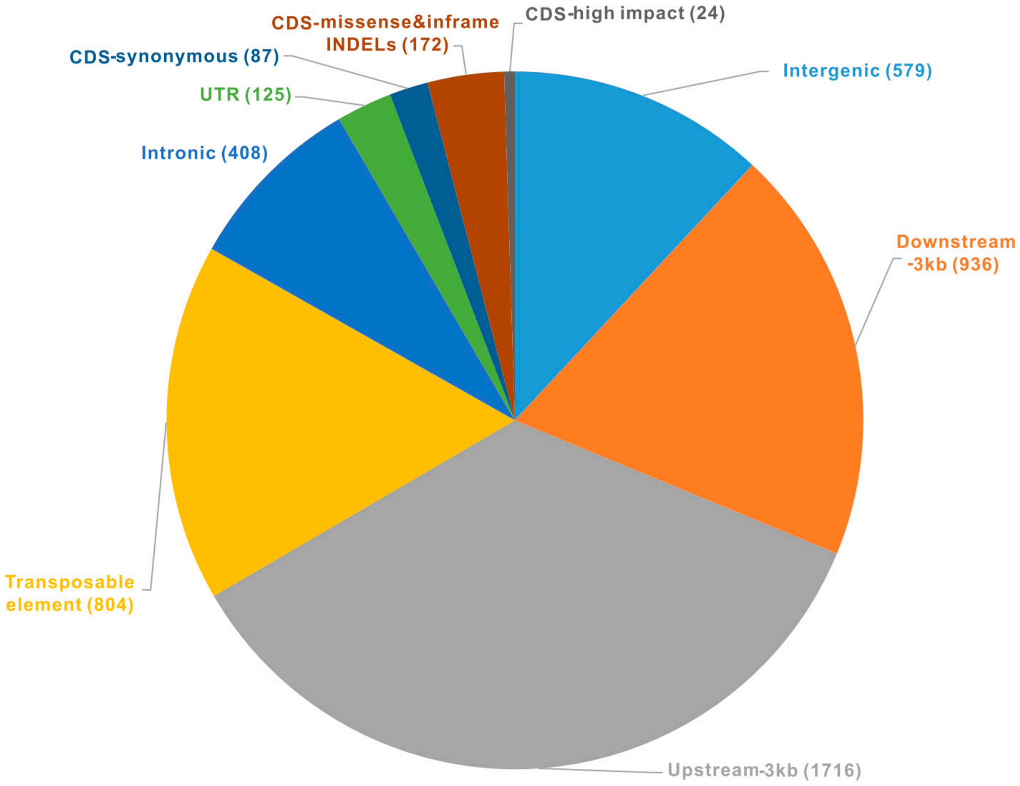

Figure S4. Comparing of encoded proteins of three *RGA1* alleles.

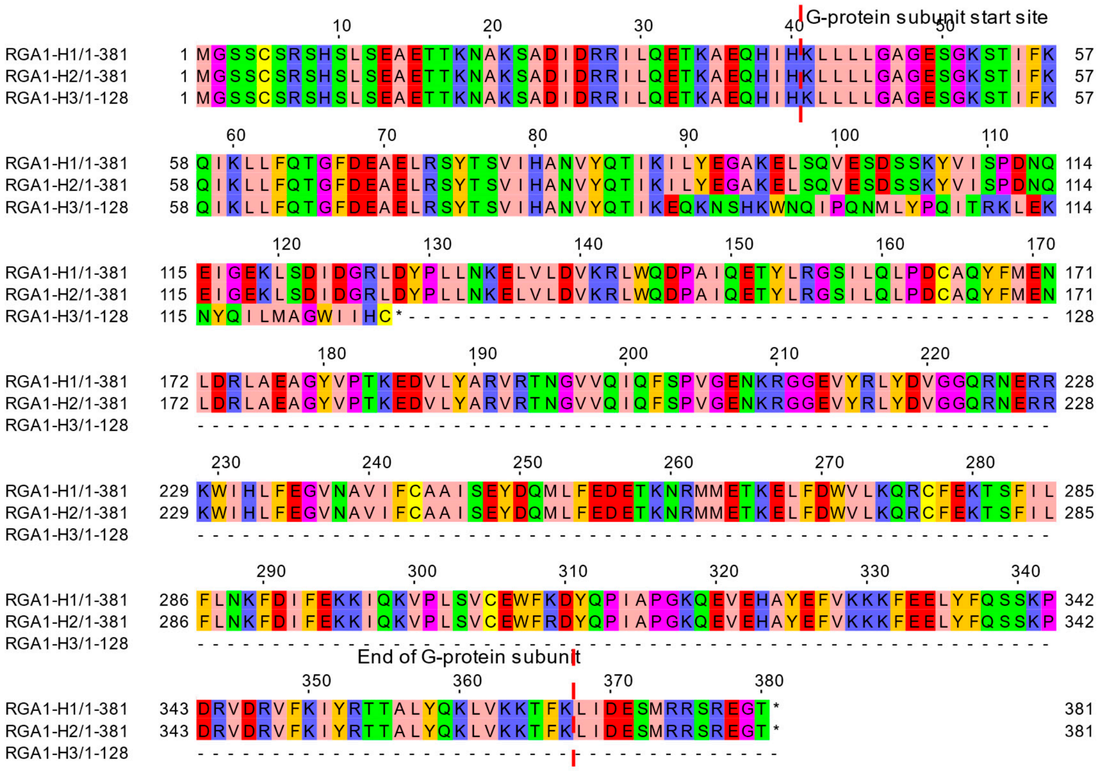

* *RGA1*-H1 and *RGA1*-H2 correspond to haplotype in Figure 2e, respectively. *RGA1*-H3 represents the predicted protein sequence of *RGA1-FH*.

Figure S5. Comparing cDNA Sequences of *RGA1* between Large and Small Grains in F2 Population.

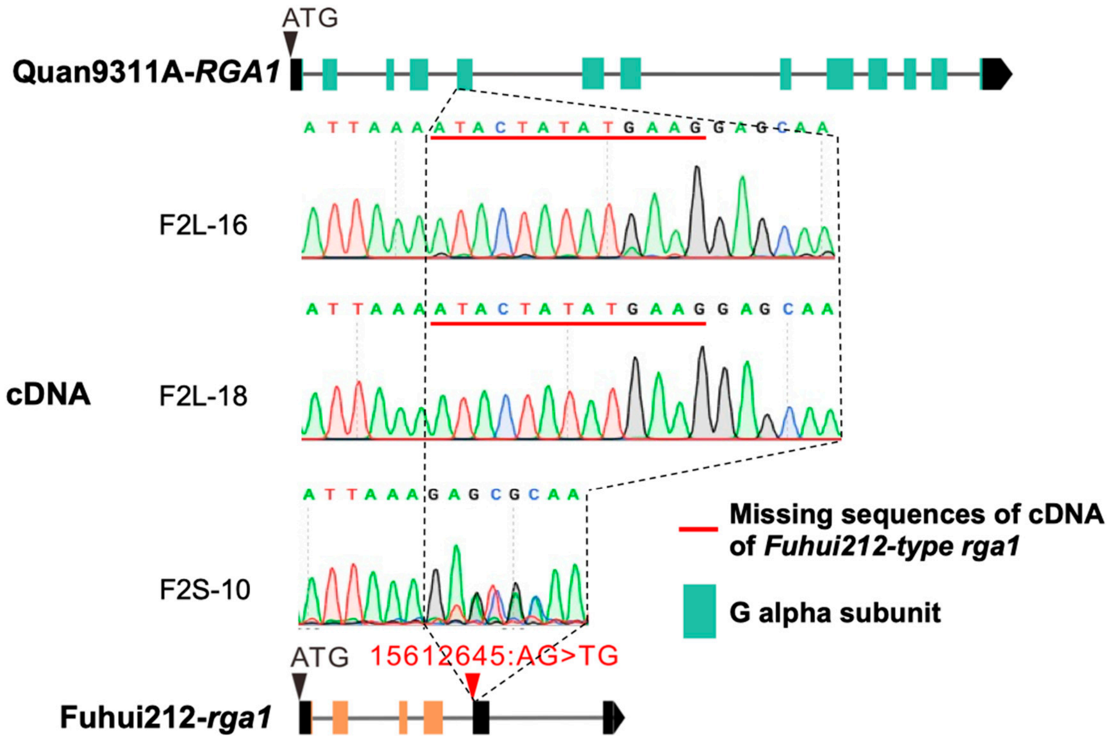

Figure S6. Expression verification of *RGA1* in transgenic lines

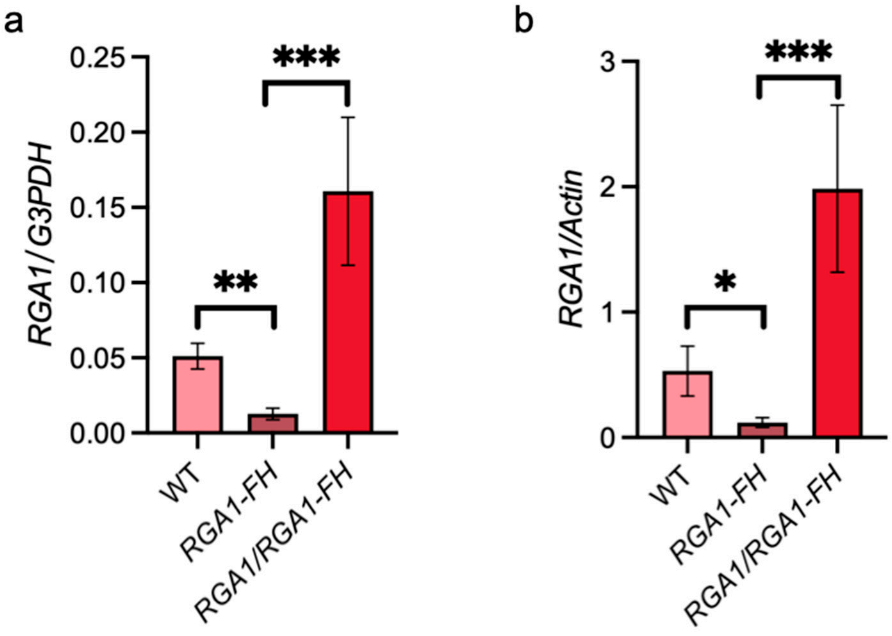

Relative expression levels of *RGA1* in large grain individuals (WT), small grains individuals (*RGA1-FH*) and transgenic lines (*RGA1/RGA1-FH*) which compared to internal reference genes *G3PDH* (a) and *Actin* (b). Statistic tests using Welch's t-test, *P < 0.05, **P < 0.01, ***P < 0.001.

Table S1. Information of primers in this study.

Primer name	Forward primer (5’ to 3’)	Reverse primer (5’ to 3’)	Expect Size (bp)	Aims
RGA1-CDS	ATGGGCTCATCCTGTAGCA	AAGTTCCTTCCCTGGA	1143 bp	*OsRGA1* CDS cloning
RGA1-comp	*GGCGCGCC*CAGGGTTGGGAGTGTGATT	*GGCGCGCC*CGGTGGACCAGGTAACTAC	7063 bp	*OsRGA1* complete sequence cloning
RGA1-qPCR-1	*GAACTCTCACAAGTGGAATC*	*CCAACCTGCCATCAATATC*	97 bp	*OsRGA1* expression identification
RGA1-qPCR-3	*TGATGAGGCAGAACTTAGG*	*CCAACCTGCCATCAATATC*	182 bp	*OsRGA1* expression identification
G3PDH-qPCR	*CATCATTCCCAGCAGCAC*	*GCAGCCTTCTCGATTCTAAC*	144 bp	*qPCR* primers of internal reference genes *G3PDH*
Actin-qPCR	*TGTATGCCAGTGGTCGTACCA*	*CCAGCAAGGTCGAGACGAA*	121 bp	*qPCR* primers of internal reference genes *Actin*

Note: AscI restriction sites are indicated in italics.

Table S2. Measured grain traits of F2 population.

Sample ID	1000 GW (g)	Grain length mean (mm)	Grain length SD	Grain width mean (mm)	Grain width SD
F2L-1	25.525	9.522	0.422	2.515	0.201
F2L-2	25.602	8.898	0.486	2.667	0.230
F2L-3	27.261	9.179	0.437	2.636	0.189
F2L-4	26.331	9.057	0.476	2.767	0.228
F2L-5	24.368	9.040	0.567	2.485	0.219
F2L-6	26.248	9.411	1.058	2.522	0.229
F2L-7	27.452	9.612	0.427	2.585	0.184
F2L-8	24.161	9.080	0.357	2.453	0.221
F2L-9	28.432	9.537	0.478	2.663	0.193
F2L-10	23.839	8.847	0.432	2.544	0.244
F2L-11	25.475	8.830	0.434	2.548	0.195
F2L-12	29.235	9.567	0.442	2.734	0.228
F2L-13	25.880	9.217	0.619	2.665	0.238
F2L-14	23.374	8.963	0.434	2.576	0.192
F2L-15	26.825	9.310	0.407	2.607	0.224
F2L-16	24.167	8.889	0.608	2.601	0.205
F2L-17	26.218	9.021	0.465	2.626	0.216
F2L-18	21.726	8.632	0.376	2.578	0.188
F2L-19	27.879	9.273	0.515	2.612	0.226
F2L-20	25.244	8.806	0.436	2.642	0.208
F2L-21	24.412	8.990	0.464	2.505	0.239
F2L-22	24.750	8.952	0.504	2.512	0.220
F2L-23	25.217	8.978	0.536	2.616	0.266
F2L-24	26.303	9.109	0.403	2.519	0.222
F2L-25	26.681	9.161	0.397	2.661	0.228
F2L-26	24.139	8.920	0.546	2.477	0.252
F2L-27	26.793	9.357	0.330	2.693	0.209
F2L-28	26.209	9.048	0.574	2.675	0.456
F2L-29	25.740	8.859	0.425	2.659	0.254
F2L-30	24.263	8.633	0.466	2.574	0.203
F2L-31	26.521	9.535	0.701	2.634	0.221
F2L-32	24.873	9.098	0.367	2.512	0.219
F2S-1	11.333	6.337	0.959	2.701	0.470
F2S-2	15.077	6.235	0.438	2.820	0.202
F2S-3	13.846	6.214	0.379	2.687	0.203
F2S-4	13.500	6.085	0.370	2.730	0.167
F2S-5	13.000	6.098	0.960	2.853	0.494
F2S-6	12.642	6.209	0.337	2.613	0.212
F2S-7	15.824	6.199	0.377	2.802	0.184
F2S-8	13.836	6.004	0.453	2.686	0.211
F2S-9	15.672	6.733	0.363	2.828	0.207

F2S-10	12.139	6.448	0.387	2.747	0.170
F2S-11	14.932	6.092	0.371	2.755	0.195
F2S-12	13.788	6.198	0.376	2.756	0.210
F2S-13	13.894	5.978	0.410	2.520	0.168
F2S-14	15.839	6.195	0.502	2.749	0.191
F2S-15	17.661	6.687	0.480	2.844	0.209
F2S-16	14.589	6.417	0.361	2.783	0.172
F2S-17	14.185	6.382	0.520	2.793	0.185
F2S-18	13.579	5.783	0.478	2.708	0.227
F2S-19	14.333	6.310	0.405	2.761	0.223
F2S-20	14.877	6.118	0.384	2.717	0.151
F2S-21	15.473	6.302	0.382	2.803	0.205

Table S3. Sequencing, mapping metrics and genotyping RGA1 of F2 samples.

Sample ID	Trait group	No. of read pairs	Clean data size (G)	Sequencing depth	Mean mapped depth with mapping quality ≥ 20	RGA1 haplotype
F2L-1	Large grain	57657939	17.3	46.4	35.03	H2/H3
F2L-10	Large grain	58614491	17.6	47.1	35.23	H2
F2L-11	Large grain	57702920	17.3	46.4	35.63	H2
F2L-12	Large grain	54391042	16.3	43.7	33.81	H2/H3
F2L-13	Large grain	53999433	16.2	43.4	33.09	H2/H3
F2L-14	Large grain	56993301	17.1	45.8	35.14	H2
F2L-16	Large grain	62172588	18.7	50.0	38.15	H2/H3
F2L-17	Large grain	57201807	17.2	46.0	33.97	H2
F2L-18	Large grain	55871400	16.8	44.9	34.1	H2
F2L-19	Large grain	61283267	18.4	49.3	37.7	H2/H3
F2L-2	Large grain	63098655	18.9	50.7	38	H2/H3
F2L-20	Large grain	43539341	13.1	35.0	26.77	H2/H3
F2L-21	Large grain	55954402	16.8	45.0	33.97	H2
F2L-22	Large grain	58597061	17.6	47.1	35.71	H2/H3
F2L-23	Large grain	57092258	17.1	45.9	34.73	H2/H3
F2L-24	Large grain	51529133	15.5	41.4	31.66	H2
F2L-25	Large grain	65178812	19.6	52.4	39.77	H2/H3
F2L-26	Large grain	56021263	16.8	45.1	33.93	H2/H3
F2L-28	Large grain	42267508	12.7	34.0	25.66	H2
F2L-29	Large grain	61624639	18.5	49.6	37.89	H2
F2L-3	Large grain	52334217	15.7	42.1	32.17	H2/H3
F2L-30	Large grain	58703876	17.6	47.2	35.93	H2
F2L-31	Large grain	56710866	17.0	45.6	33.84	H2/H3
F2L-32	Large grain	68316360	20.5	54.9	40.19	H2/H3
F2L-4	Large grain	58237488	17.5	46.8	35.38	H2
F2L-5	Large grain	58424718	17.5	47.0	35.79	H2/H3
F2L-6	Large grain	50325592	15.1	40.5	30.53	H2/H3
F2L-7	Large grain	58604420	17.6	47.1	35.48	H2/H3
F2L-8	Large grain	62313010	18.7	50.1	38.26	H2/H3
F2L-9	Large grain	56874861	17.1	45.7	34.86	H2/H3
F2S-1	Small grain	54571081	16.4	43.9	33.53	H3
F2S-10	Small grain	53118373	15.9	42.7	32.51	H3
F2S-12	Small grain	58143745	17.4	46.8	35.69	H3
F2S-13	Small grain	59612797	17.9	47.9	36.57	H3
F2S-14	Small grain	56172082	16.9	45.2	34.14	H3
F2S-15	Small grain	48883846	14.7	39.3	30.07	H3
F2S-16	Small grain	47696234	14.3	38.4	29.03	H3
F2S-17	Small grain	41235410	12.4	33.2	25.27	H3
F2S-18	Small grain	32980167	9.9	26.5	20.22	H3

F2S-19	Small grain	47808781	14.3	38.5	29.08	H3
F2S-2	Small grain	44714216	13.4	36.0	27.38	H3
F2S-20	Small grain	36730908	11.0	29.5	22.45	H3
F2S-21	Small grain	43223715	13.0	34.8	26.63	H3
F2S-4	Small grain	43061393	12.9	34.6	26.49	H3
F2S-5	Small grain	50031634	15.0	40.2	30.76	H3
F2S-6	Small grain	44033012	13.2	35.4	27.03	H3
F2S-7	Small grain	55073111	16.5	44.3	33.8	H3
F2S-8	Small grain	56110773	16.8	45.1	34.22	H3
F2S-9	Small grain	39164045	11.7	31.5	24.04	H3

Note: The RGA1 haplotype number in this table corresponds to the number in Figure 2e and Figure S4.

Table S4. Candidate QTL regions with genome-scan-adjusted p-value ≤ 0.05.

Candidate QTL region	Genome-scan-adjusted p-values	No. of non-TE protein-coding genes	No. of TEs
chr01:35574787-35574913	0.01	0	1
chr02:23341009-23341034	0.01	0	1
chr05:3136610-3370587	0.01	30	2
chr05:3427622-3430304	0.01	1	0
chr05:3432976-3633726	0.01	24	5
chr05:3651690-4094550	0.01	39	18
chr05:4114609-4349880	0.01	28	8
chr05:4404085-4411800	0.05	1	0
chr05:4538528-4726058	0.05	29	4
chr05:4749657-4850242	0.01	10	3
chr05:4853242-4913532	0.01	7	2
chr05:4914327-5010193	0.05	11	4
chr05:5031176-5659773	0.01	58	27
chr05:5780174-5833311	0.01	5	2
chr05:6014588-6579257	0.01	55	17
chr05:6604425-6857523	0.01	26	5
chr05:6858559-7045765	0.01	25	5
chr05:7048430-7093295	0.01	4	3
chr05:7098163-7098670	0.01	0	1
chr05:7104594-7361033	0.01	28	15
chr05:7363012-7485970	0.01	14	6
chr05:7491796-7539272	0.01	6	2
chr05:7547808-7717831	0.01	20	9
chr05:7747060-7857515	0.01	8	5
chr05:7879296-8052834	0.01	17	3
chr05:8058277-8145611	0.01	3	9
chr05:8147890-8217467	0.01	4	3
chr05:8218083-8326024	0.01	4	3
chr05:8326080-8342705	0.01	0	6
chr05:8344315-8559766	0.01	26	9
chr05:8563749-8618865	0.01	8	3
chr05:8642802-8748611	0.01	8	14
chr05:8750587-8790219	0.01	3	2
chr05:8805394-8898520	0.01	11	10
chr05:8898631-8986295	0.01	10	5
chr05:8987062-9016810	0.01	0	4
chr05:9017321-9025611	0.01	0	1
chr05:9031022-9082678	0.01	5	4
chr05:9093782-9155626	0.01	8	2
chr05:9155823-9173692	0.01	0	1
chr05:9174012-9271024	0.01	8	8

chr05:9278542-9284344	0.01	0	1
chr05:9287402-9352329	0.01	5	4
chr05:9352555-9402169	0.01	2	9
chr05:9404908-9712988	0.01	15	24
chr05:9727512-9850576	0.01	0	16
chr05:9870454-10248106	0.01	7	45
chr05:10271884-10380858	0.01	1	19
chr05:10415168-10498548	0.01	2	12
chr05:10522625-10604640	0.01	3	8
chr05:10637981-10700110	0.01	1	7
chr05:10715009-10888556	0.01	11	10
chr05:10898822-11093856	0.01	17	18
chr05:11114459-11132016	0.01	1	3
chr05:11144744-11449899	0.01	16	27
chr05:11470302-11559460	0.01	2	13
chr05:11571204-11727079	0.01	11	9
chr05:11746062-11962128	0.01	5	24
chr05:11971983-12006893	0.01	1	6
chr05:12018749-12052743	0.01	0	5
chr05:12092644-12100404	0.01	0	1
chr05:12118021-12497492	0.01	7	49
chr05:12573832-13365487	0.01	45	69
chr05:13392800-13529365	0.01	11	4
chr05:13555489-13691964	0.01	8	8
chr05:13715502-14229265	0.01	30	43
chr05:14235366-14533368	0.01	22	24
chr05:14534415-14888616	0.01	32	18
chr05:14891737-15097388	0.01	29	7
chr05:15103962-15127504	0.01	1	2
chr05:15135688-15199495	0.01	5	5
chr05:15200689-15291916	0.01	8	11
chr05:15299461-15326111	0.01	0	5
chr05:15412316-15544309	0.01	10	19
chr05:15552453-15623176	0.01	8	1
chr05:15631320-15664177	0.01	3	0
chr05:15665600-15730949	0.01	5	3
chr05:15741489-15788795	0.01	4	1
chr05:15790840-15838685	0.01	2	7
chr05:15839181-15955285	0.01	8	11
chr05:15990607-16052045	0.01	8	2
chr05:16134727-16221709	0.01	8	4
chr05:16255129-16985735	0.01	73	28
chr05:17012997-17048563	0.01	3	0
chr05:17068183-17223434	0.01	11	14

chr05:17240604-17283869	0.01	5	3
chr05:17300992-17370442	0.01	10	1
chr05:17418272-17745617	0.01	35	18
chr05:17748559-18118031	0.01	44	7
chr05:18149518-18278547	0.01	12	8
chr05:18290380-18326426	0.01	4	2
chr05:18332912-18377736	0.01	5	2
chr05:18381927-18401602	0.01	1	3
chr05:18404421-18766176	0.01	39	14
chr06:18326998-18447401	0.01	1	23
chr07:21947959-21948528	0.05	0	1
chr12:7785337-7786706	0.01	0	1

Table S5. Genes with large impact variants segregated between large grain and small grain plants. Effects predicted based on MSU v7 annotations.

Locus ID	Gene description	CDS change	Variant position in CDS	Predicted effect	Predicted malfunctional genotype
LOC_Os05g25670	expressed protein	T>C	298	stop lost	Quan9311A
LOC_Os05g25760	expressed protein	C>T	316	stop gained	Quan9311A
		G>A	533	stop gained	Quan9311A
LOC_Os05g25830	expressed protein	INS: GAACAGAGAGAAGCACAGGGGA	1090	frameshift variant	Quan9311A
		C>T	712	stop gained	Quan9311A
		DEL: G	247	frameshift variant	Quan9311A
		C>T	157	stop gained	Quan9311A
LOC_Os05g25890	expressed protein	INS: TGGT	832	frameshift variant	Quan9311A
LOC_Os05g25930	expressed protein	DEL: A	351	frameshift variant	Quan9311A
LOC_Os05g25990	expressed protein	DEL: G	720	frameshift variant	Fuhui212
		DEL: AGGCGGTGGACGCGGCGAGGAGGGC	677	frameshift variant	Quan9311A
		C>T	340	stop gained	Quan9311A

LOC_Os05g26040	pumilio-family RNA binding repeat containing protein, expressed	DEL: ACCGCCCCACCACGGGAGA	213	frameshift variant	Quan9311A
		DEL: AA	1706	frameshift variant	Quan9311A
		DEL: G	3829	frameshift variant	Quan9311A
LOC_Os05g26070	cyclin, putative, expressed	A>G	1	start lost	Quan9311A
LOC_Os05g26210	ATOFP17/OFP1, putative, expressed	DEL: TTAG	698	frameshift variant	Quan9311A
LOC_Os05g26850	hypothetical protein	INS: TTGAAACGTAAAGACAGATGGAGATATTG	142	frameshift variant	Quan9311A
		C>T	94	stop gained	Fuhui212
LOC_Os05g26860	expressed protein	DEL: A	519	frameshift variant	Quan9311A
LOC_Os05g26870	CAMK_KIN1/SNF1/Nim1_like.21 - CAMK includes calcium/calmodulin dependent protein kinases, expressed	DEL: TCTC	153	frameshift variant	Quan9311A
		INS: TATC	129	frameshift variant	Quan9311A
LOC_Os05g26890	D1/RGA1, G-protein alpha	A>T	154*	splice acceptor variant	Fuhui212

	subunit, putative, expressed				
LOC_Os05g26980	expressed protein	DEL: G	108	frameshift variant	Fuhui212

*position refers to the start position of intron adjacent to exon (i.e., 153bp coding + 1bp intron)

Table S6 Phenotypic statistics of grains of wild-type F2L-30, *RGA1-FH* type F2S-16 and the complemental lines.

	WT	*RGA1-FH*	*RGA1/RGA1-FH*
Thousand Grain weight (g)	19.930	15.819	17.732
Mean length/width	3.492	2.433	3.478
Mean length (± SD) (mm)	8.865 ± 0.822	6.802 ± 0.693	8.885 ± 0.898
Mean width (± SD) (mm)	2.589 ± 0.295	2.840 ± 0.305	2.604 ± 0.328
Diameter (mm)	4.849	4.379	4.844
Mean roundness	0.297	0.428	0.298
